# Epithelioid sarcoma with multiple lesions on the left arm: a case report

**DOI:** 10.1186/s13256-016-1088-z

**Published:** 2016-10-24

**Authors:** Rie Nishibaba, Yuko Higashi, Yuko Goto, Masanori Hisaoka, Takuro Kanekura

**Affiliations:** 1 Department of Dermatology, Kagoshima University Graduate School of Medical and Dental Sciences, 8-35-1 Sakuragaoka, Kagoshima, 890-8520 Japan; 2 Department of Pathology, Field of Oncology, Kagoshima University Graduate School of Medical and Dental Sciences, Kagoshima, Japan; 3 Department of Pathology and Oncology, School of Medicine, University of Occupational and Environmental Health, Kitakyusyu, Japan

**Keywords:** CA-125, Epithelioid sarcoma, E26-related gene, Integrase interactor 1, Malignant soft tissue tumor

## Abstract

**Background:**

Epithelioid sarcoma is a rare, high-grade malignant tumor of the soft tissue. The incidence of local recurrence, regional lymph node involvement, and distant metastases is high. Epithelioid sarcoma is most often seen in adolescents and young adults. In the early stage before the development of full clinical features, epithelioid sarcoma is often misdiagnosed as a benign disease such as granuloma.

**Case presentation:**

We report a case of a 74-year-old Japanese woman whose epithelioid sarcoma was initially misdiagnosed as fungal infection. Rebiopsy revealed the proliferation of atypical polygonal or oval epithelioid cells in the dermis and lymphocyte infiltration through the dermis. Immunohistochemically, the tumor cells were positive for vimentin, cell adhesion molecule 5.2, epithelial membrane antigen, and E26-related gene. The nuclear expression of integrase interactor 1 was lost in the tumor cells.

**Conclusions:**

We encountered a rare case of epithelioid sarcoma and had difficulty in making the correct diagnosis. We suggest that in patients whose lesions are resistant to conventional treatments, repeat biopsy and immunohistochemical studies should be considered to rule out rare epithelioid sarcoma.

## Background

Epithelioid sarcoma (ES), first described by Enzinger in 1970 [1], is a rare sarcoma localized to deep or superficial soft tissue. It is the most common soft tissue sarcoma of the hand and wrist, followed by alveolar rhabdomyosarcoma and synovial sarcoma. ES can develop at any age but is rarely seen in children and the elderly. Because it is very rare and appears to be a benign lesion in the early stage, ES is often misdiagnosed as a benign disease such as granuloma. We report a case of a patient with ES on the hand and forearm with multiple satellite cutaneous lesions.

## Case presentation

**Fig. 1:**
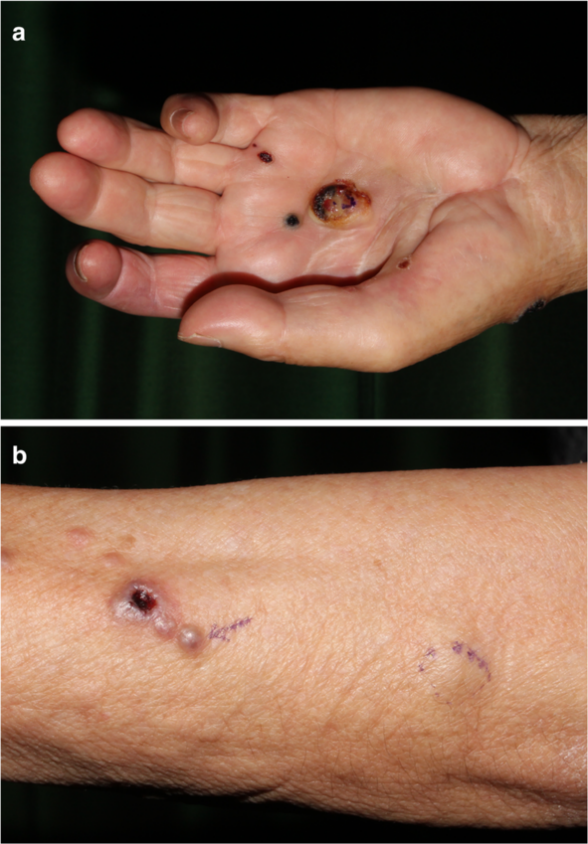
**a** A 2-cm ulcerated nodule on the left hand. **b** Multiple lesions on the patient’s left forearm

**Fig. 2:**
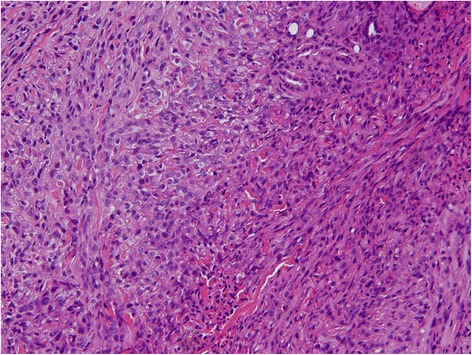
Histopathological examination of a skin biopsy from a nodule on the palm. The lesion was composed of a proliferation of atypical polygonal or oval epithelioid cells in the dermis with lymphocytic infiltration through the dermis. Hematoxylin and eosin stain, original magnification ×200

**Fig. 3:**
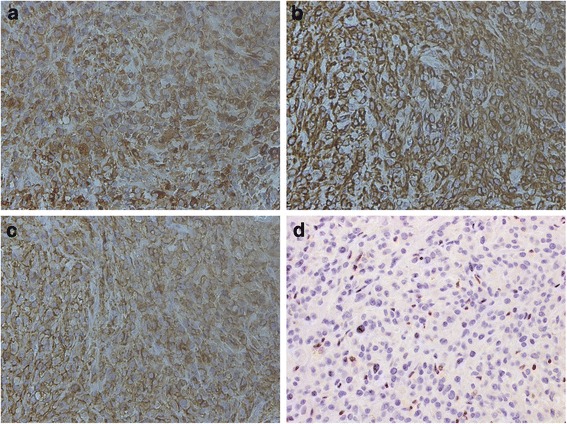
Immunohistochemical staining for (**a**) vimentin, (**b**) cell adhesion molecule 5.2, and (**c**) epithelial membrane antigen. (**d**) The nuclear expression of integrase interactor 1 was diminished in the tumor cells

A 74-year-old Japanese woman presented to our hospital with multiple ulcerated nodules on the left hand and forearm. The nodules had slowly increased in size and number for 2 months. An examination revealed a 2-cm ulcerated nodule on her left hand; she had multiple satellite lesions on her left hand and forearm (Fig. 1a, b). The initial differential diagnoses included subcutaneous mycosis and malignant melanoma. Histopathological examination showed ulceration and granulomatous lesions with multinucleated giant cells in the deep dermis. A periodic acid-Schiff stain revealed no fungi in the specimen, but a tissue culture was positive for *Alternaria*, a dematiaceous fungus. A diagnosis of dermal cutaneous alternariosis was made. The patient was started on itraconazole therapy, but her nodules increased in size and number within the next 2 months. Rebiopsy of a nodule on the patient’s palm revealed the proliferation of atypical polygonal or oval epithelioid cells in the dermis and lymphocyte infiltration through the dermis (Fig. 2). Immunohistochemically, the tumor cells were positive for vimentin, cell adhesion molecule 5.2 (CAM5.2), epithelial membrane antigen (EMA), and E26-related gene (ERG) (Fig. 3a–c); they were negative for AE1/AE3, CD34, S100, smooth muscle actin, and CD31. The nuclear expression of integrase interactor 1 (INI1) was lost in the tumor cells (Fig. 3d).

**Fig. 4:**
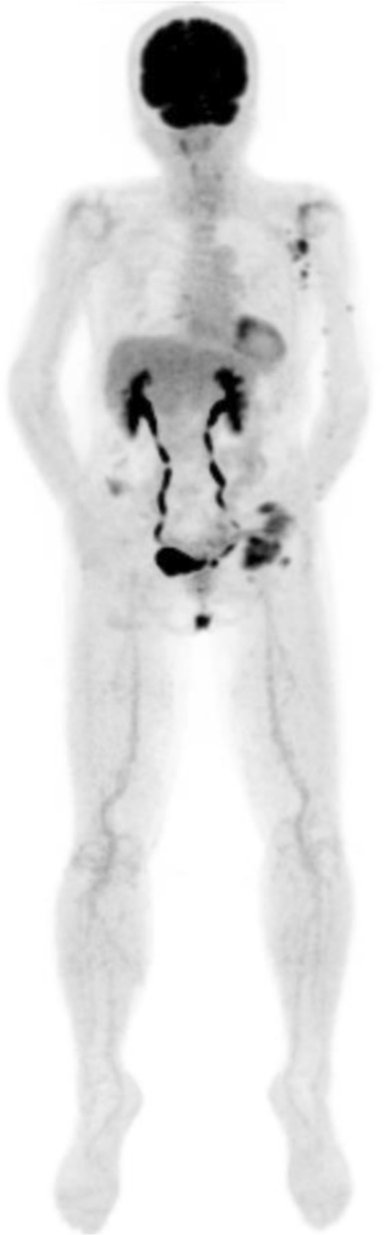
Preoperative fluorodeoxyglucose-positron emission tomography showed elevated glucose levels in the multiple lesions on the *left* hand, *left* forearm, and *left* axillary lymph nodes

On the basis of these findings, a diagnosis of ES was made. Preoperative fluorodeoxyglucose-positron emission tomography showed elevated glucose levels in the multiple lesions on the patient’s left hand, left forearm, and left axillary lymph nodes (Fig. 4). After shoulder disarticulation and axillary lymph node dissection, there was no recurrence, and no metastases were observed during 9 months of follow-up.

## Discussion

ES, a rare and highly malignant soft tissue tumor, shows a high tendency for local recurrence, regional lymph node involvement, and distant metastases [1]. ES constitutes less than 1 % of all soft tissue sarcomas [2]. It is characterized clinically by a firm and slow-growing tumor with a predilection for the hands, fingers, and forearms. Fifty-four percent of cases arise in the distal upper extremities [2]. Pain or tenderness is rarely a prominent symptom, unless large nerves are affected by the tumor. ES is commonly seen in young patients; the peak incidence is in the third decade of life, and it is rare in children and the elderly.

Recurrence and metastasis are reported in 77 % and 45 % of patients with ES, respectively. The most common sites of metastasis are the lungs (51 %), regional lymph nodes (34 %), scalp (22 %), and bone (13 %) [2]. ES is highly malignant; 32 % of patients die as a result of the disease [3]. Male sex, proximal location, large tumor size (greater than 50 mm), deep location, high mitotic index, hemorrhage, necrosis, vascular invasion, and inadequate initial excision causing local recurrence have been shown to be poor prognostic factors [4]. Clinical features of the reported cases are summarized in Table 1.

**Table 1:** Clinical features of previously reported cases and our patient

	Reported cases [5]	Our patient
Male sex, %	42–79	−
Female sex, %	21–62	+
Mean age at first symptoms, years	23–41	74
Mean symptom duration before diagnosis, months	7.1–36	4
Local recurrence, %	29–85	+
Lymph node metastases, %	11–65	+
Distant metastases, %	21–62.5	−

A new subtype, a proximal variant of ES, has been proposed [5]. This subtype exhibits more aggressive clinical behavior, and its histopathological appearance is distinct. Clinically, the proximal subtype differs from the classical type by its multinodular growth pattern, its more frequent occurrence in older patients, its more proximal/axial distribution, its more deep-seated location, and its more aggressive clinical behavior from the outset. Histologically, it is characterized by predominantly large cells, epithelioid cytomorphology, marked cytological atypia, the frequent manifestation of rhabdoid features, and the absence of a granuloma-like pattern in most patients. On the basis of our clinical and histological findings, we considered the ES in our patient to be of the conventional distal type.

The cellular elements of ES range from large ovoid or polygonal cells with deeply eosinophilic cytoplasm to plump, spindle-shaped cells [3]. Consequently, ES is often misdiagnosed as one of numerous benign and malignant disorders, and proper treatment is delayed. The median interval between the initial diagnosis and the start of treatment is 3.5 months (range 1–6 months) [6]. Nishimura *et al.* [7] reported a patient with ES masquerading as an intractable wound for more than 18 years. We correctly diagnosed our patient 4 months after her initial presentation.

Immunohistochemical examination is essential to making a diagnosis of ES. Characteristically, it shows expression of vimentin and low-molecular-weight cytokeratins such as those recognized by the monoclonal antibody CAM5.2 [8, 9], and 85–96 % of cases are EMA-positive [4]. The staining pattern suggests that ES is a neoplasm of mesenchymal origin with epithelial dedifferentiation. Vimentin is rarely negative. CD34, expressed in approximately 50 % of ES lesions, is used as an additional diagnostic marker in vimentin-negative cases. The *INI1* gene is a member of the adenosine triphosphate-dependent SWI/SWF chromatin-remodeling complex and thus a candidate as a tumor suppressor gene. Loss of the *INI1* gene is seen in some patients with malignant rhabdoid tumors; its loss is 93 % sensitive for a diagnosis of ES [10].

ERG, a member of the erythroblast transformation-specific family of transcription factors, is a marker of endothelial differentiation [11]. Kohashi *et al.* [12], who analyzed the expression of ERG in *INI1*-deficient tumors, including ES, reported that ERG was expressed in 13 of 24 conventional distal type lesions and 5 of 20 patients with proximal-type ES. All of their patients with malignant rhabdoid tumors had negative test results for ERG, suggesting that ERG may be a useful marker to distinguish ES from malignant rhabdoid tumors with loss of *INI1*.

Cancer antigen 125 (CA-125) is a high-molecular-weight glycoprotein commonly used for the identification of epithelial ovarian carcinomas; it has also been detected in various neoplasms, including carcinomas of the breast, lung, and colon, and in patients with lymphoma. Kato *et al.* [13] documented that 10 (91 %) of 11 patients with ES had positive test results for CA-125; 10 manifested the classical type and the other had the proximal type of the disease. In other soft tissue tumors, including synovial sarcomas, clear cell sarcomas, leiomyosarcomas, rhabdomyosarcomas, liposarcomas, malignant peripheral nerve sheath tumors, malignant fibrous histiocytomas, desmoid tumors, cutaneous squamous cell carcinomas, and rheumatoid nodules, CA-125 was negative. Other authors [8, 9] have suggested that CA-125 immunoreactivity in the presence of an elevated serum CA-125 level could be a useful tumor marker for the diagnosis of ES and for monitoring the clinical course of patients with ES.

## Conclusions

ES is very rare and appears to be a benign lesion in the early stage, and it is often misdiagnosed as a benign disease such as granuloma. We encountered a rare case of ES and had difficulty in making the correct diagnosis. We suggest that in patients whose lesions are resistant to conventional treatments, repeat biopsy and immunohistochemical studies should be considered to rule out rare ES.
